# Juvenile Hamartomatous Polyp Causing Jejunal Intussusception in an Eight-Year-Old Child: A Case Report

**DOI:** 10.7759/cureus.100517

**Published:** 2025-12-31

**Authors:** Gustavo Sugai, Eduardo Motta Buchaim, Tiago Mestriner Costa, Beatriz Gordilho Bacos, Felipe Paglioli

**Affiliations:** 1 Clinical Medicine, Irmandade de Misericórdia da Santa Casa de São Paulo, São Paulo, BRA; 2 Internal Medicine, Irmandade de Misericórdia da Santa Casa de São Paulo, São Paulo, BRA; 3 Internal Medicine, Universidade de Medicina Santo Amaro, São Paulo, BRA

**Keywords:** chronic abdominal pain, colon cancer and colon polyps, gastrointestinal obstruction, hamartomatous polyp, polyps

## Abstract

While juvenile hamartomatous polyps are common pediatric findings, they are almost exclusively restricted to the colon and rectum; their occurrence in the small intestine is exceptionally rare and presents a significant diagnostic dilemma. This case report describes an eight-year-old patient who presented with vague constitutional symptoms, including intermittent vomiting, abdominal pain, and severe weight loss resulting in a Z-score of -3.29. Initial diagnostic investigations, including abdominal ultrasound, computed tomography, upper digestive endoscopy, and contrast bowel studies, failed to identify a clear etiology. Following an exploratory laparotomy, a juvenile hamartomatous polyp was identified in the mid-jejunum, acting as a lead point for intermittent intussusception with secondary vascular congestion of the intestinal loops. The diagnosis was confirmed through anatomopathological analysis after a successful enterectomy. This case demonstrates that in the presence of unexplained malnutrition and cyclical emesis, clinicians must maintain a high index of suspicion for small bowel lesions, as prompt surgical intervention can prevent further complications and lead to complete symptomatic resolution.

## Introduction

Juvenile hamartomatous polyps (JP) are the most frequent type of gastrointestinal polyp in the pediatric population, accounting for approximately 90% of cases. These lesions are almost exclusively found in the lower gastrointestinal tract, specifically in the colon and rectum. In contrast, the occurrence of a solitary juvenile polyp in the small intestine, particularly the jejunum, is exceptionally rare and significantly less documented, which complicates clinical and surgical management [1].

Gastrointestinal polyps are defined as epithelial or submucosal nodules that project into the intestinal lumen. While histological characteristics dictate their classification as benign or malignant, juvenile polyps are primarily characterized by an edematous lamina propria and dilated cystic glands [1]. Commonly, juvenile polyps present with intestinal bleeding and abdominal pain; however, these symptoms are often vague and overlap with other abdominal syndromes [2]. Standard diagnostic protocols typically involve endoscopy, barium enema, or colonoscopy, which serves as the gold standard for both diagnosis and removal, followed by anatomopathological examination [3].

However, unlike colonic lesions, jejunal polyps reside in a segment often inaccessible to standard upper digestive endoscopy and colonoscopy. In the case detailed herein, this anatomical location hindered the diagnostic process for a patient presenting with intermittent intestinal obstruction, vomiting, and severe malnutrition. Initial investigations - including abdominal ultrasound, computed tomography, upper digestive endoscopy, and contrast studies - failed to identify the pathology. These inconclusive findings led to a prolonged hospital stay before a definitive diagnosis could be reached. This report describes a uniquely rare clinical entity, as a search of the literature revealed no cases with a similar symptomatic presentation and location.

## Case presentation

An eight-year-old male patient presented with a five-month history of intermittent vomiting, abdominal pain, and a weight loss of 5 kg. Clinical assessment at the time of admission revealed severe malnutrition, evidenced by a body mass index (BMI) of 12 and a Z-score of -3.29, placing the patient well below the first percentile for his age group. Physical examination noted a hollow abdomen with active bowel sounds, which was flaccid and painless to palpation. There was a visible loss of subcutaneous fat, though no visceromegaly, palpable masses, or signs of peritonitis were detected. The episodes of vomiting occurred twice monthly, typically lasting for two days. The patient had no relevant medical, family, or psychosocial history.

The patient was hospitalized for a comprehensive diagnostic workup due to severe malnutrition. Over a seven-day initial inpatient stay, laboratory tests (including complete blood count, albumin, and inflammatory markers) were performed and returned within normal limits. Concurrent imaging studies included abdominal ultrasound, computed tomography (CT) of the abdomen, upper digestive endoscopy, contrast studies, specifically small bowel transit and barium enema, and CT angiography to investigate potential vascular malformations. These investigations did not reveal any significant pathological changes. The patient continued to exhibit symptoms of abdominal pain and intermittent vomiting - lasting two days per episode - interspersed with asymptomatic periods. Physical examinations performed during these symptomatic crises remained unremarkable, with no palpable changes observed. Given the stability of the patient and the inconclusive nature of the extensive testing, the medical team initially opted for outpatient follow-up.

One month after the initial hospitalization and following an inconclusive diagnostic workup, the patient returned to the emergency department in a dehydrated state with persistent symptoms. Due to the prolonged period without a definitive diagnosis and the persistence of obstructive symptoms, the pediatric surgery team initially indicated a diagnostic videolaparoscopy. This procedure was subsequently converted to an exploratory laparotomy to allow for better exposure and manual palpation of the intestinal loops.

**Figure 1:**
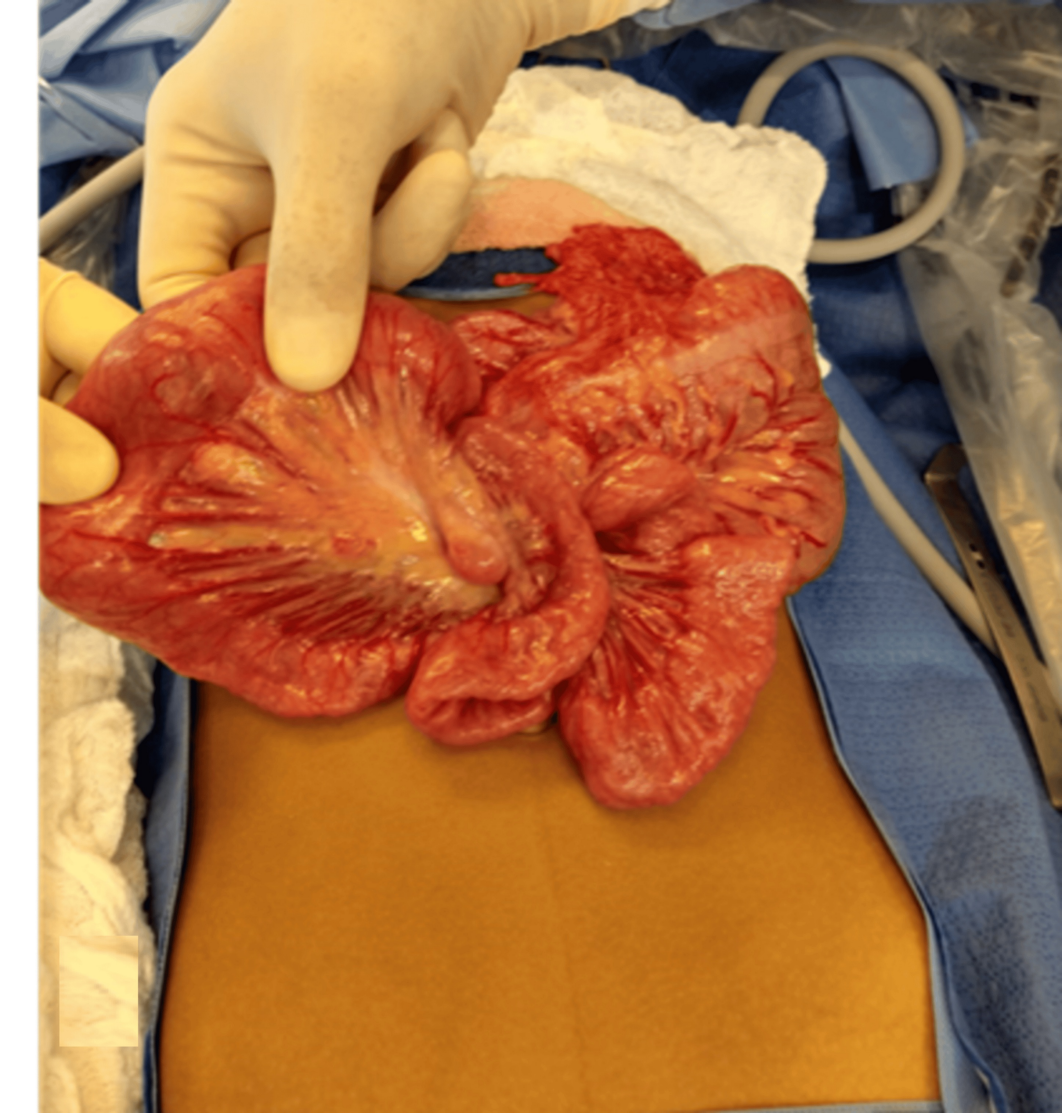
Vascular congestion of the mid-jejunum proximal to the lesion

**Figure 2:**
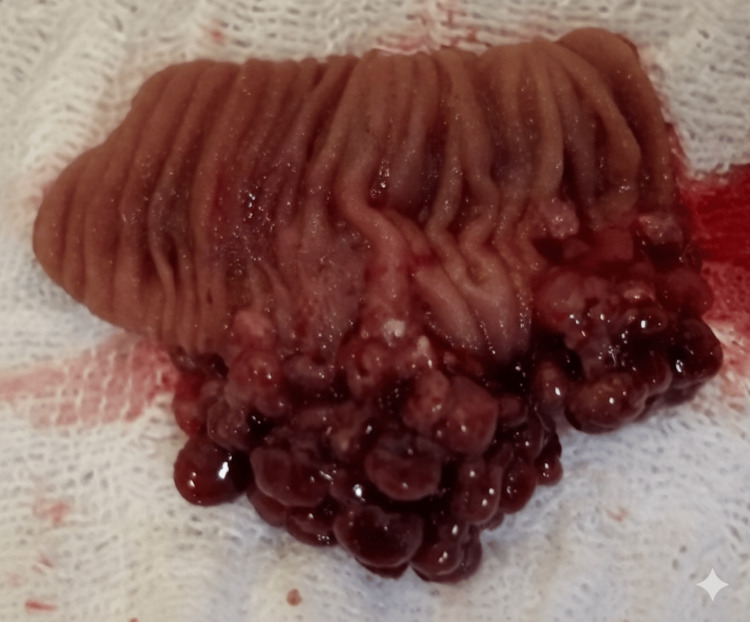
Macroscopic view of the polyp that acted as the main point for intussusception. Bowel resection showing multiple spherical pedunculated polyps with smooth surfaces.

During the surgical exploration, a mass was identified 70 mm from the angle of Treitz, accompanied by vascular congestion of the mid-jejunum proximal to the lesion (Figure 1). The surgical team performed an enterectomy of the affected segment followed by a primary anastomosis. Upon opening the resected segment, a pedunculated intraluminal polypoid mass was found (Figure 2). No other anatomical alterations were observed in the small intestine proximal or distal to the mass.

The resected material was submitted for anatomopathological analysis, which identified the lesion as a juvenile hamartomatous polyp. Histologically, the specimen was characterized by an abundance of edematous lamina propria, inflammatory cells, and characteristic dilated cystic glands lined by cubic or columnar epithelium.

Following the removal of the polyp, the patient experienced a significant resolution of symptoms and was discharged five days postoperatively. In the subsequent follow-up period, the patient remained free of vomiting and achieved significant weight gain. The patient continues to be monitored in an outpatient setting.

## Discussion

Gastrointestinal polyps are masses or nodules of epithelial or submucosal growth that project toward the lumen of the intestine and can appear in any part of the gastrointestinal tract (GIT) [1]. JP are characterized histologically by presenting an abundance of edematous lamina propria, inflammatory cells, and dilated cystic glands lined by cubic or columnar epithelium. Macroscopically, juvenile polyps range in size from 5 to 50 mm and are spherical, lobulated, and pedunculated with superficial erosion [4].

Although rare in the general population, it is the most common type of polyp in children, accounting for approximately 90% of cases [1]. In the pediatric population, it is more common for patients to have only a single juvenile polyp [1]. When more than five polyps are identified, the patient has juvenile polyposis syndrome, which, in addition to the difference in the number of polyps found, also presents neoplastic changes in the epithelium of the polyps and is associated with a higher risk of developing gastrointestinal cancers [3]. Unlike juvenile polyposis syndrome, simple juvenile polyps are lesions that are not associated with malignant transformations and are therefore considered benign. However, due to the correlation between family members with juvenile polyposis syndrome and the appearance of simple juvenile polyps in children, it is necessary to investigate the family history [3].

Regarding the location of polyps in the GIT, they are mainly found in the colon, more specifically in the rectosigmoid transition [1]. Unlike the usual presentation, the patient in this report presented the growth of the polyp in the jejunal portion of the intestine. The incidence of polyps in the jejunum is much less reported and studied than in the rectosigmoid regions, which makes their identification difficult and, consequently, the clinical and surgical management for patients with this type of lesion.

The most frequent symptoms of patients with juvenile polyps are rectal bleeding, abdominal pain, anemia, diarrhea, and constipation [3]. Rectal bleeding is the most frequent symptom [3]. In the case described, frequent vomiting was the patient's main complaint, along with abdominal pain and anemia. After laparotomy, the hypothesis for this atypical presentation was that episodes of intussusception of the intestinal loops caused by the polyp were occurring. The intussusception occurred sporadically and resolved quickly, making it difficult to identify in imaging tests and also in the patient's physical examination.

Intussusception impairs peristalsis, obstructs the passage of intestinal contents, and compromises vascular flow, leading to intestinal obstruction with secondary inflammation of the intestinal walls [5]. In this case, the juvenile hamartomatous polyp acted as the pathological lead point for intermittent intussusception.

The patient’s clinical presentation offers important pathophysiological insights. The frequent vomiting was not merely a reaction to pain but a direct neural reflex triggered by mechanical obstruction and visceral distension caused by the intussusception. Furthermore, while anemia can be influenced by local inflammatory reactions, in the context of juvenile polyps, it is classically attributed to chronic occult gastrointestinal bleeding. These polyps are macroscopically characterized by superficial erosion, which serves as a consistent source of blood loss. In this patient, this chronic hemorrhage, compounded by the nutritional impact of intermittent obstruction, drove the severe malnutrition and anemia [6].

Regarding diagnosis, standard modalities include ultrasound, CT, and endoscopy [3]. While literature suggests that CT is generally superior to ultrasound for precise localization and can reduce unnecessary surgeries, it failed to identify the lesion in this specific case [7,8]. This discrepancy likely highlights the limitations of CT in detecting small, mobile lead points during the intervals between episodes of intermittent intussusception. Additionally, while colonoscopy is considered the gold standard for the diagnosis and removal of juvenile polyps, its utility is anatomically restricted to the colon and terminal ileum [3]. The mid-jejunal location of this polyp placed it beyond the reach of both standard upper endoscopy and colonoscopy. Consequently, these standard non-invasive methods were insufficient, necessitating diagnostic videolaparoscopy with conversion to laparotomy, where the polyp was finally identified via direct palpation.

The small intestine presents a diagnostic blind spot for standard endoscopic evaluation, as significant segments remain inaccessible to conventional upper endoscopy and colonoscopy. To address this, modalities such as capsule endoscopy and enteroscopy have been developed [9].

Capsule endoscopy allows non-invasive visualization of the small bowel and is useful for screening in polyposis syndromes [10]. However, its utility in this specific clinical scenario is questionable, as it does not allow biopsy or therapeutic resection, and more importantly, it carries a risk of capsule retention in patients with suspected obstruction or stenosis [9]. Given the patient’s intermittent intussusception, this risk rendered capsule endoscopy a potentially hazardous option. Similarly, while double-balloon enteroscopy allows for tissue collection and therapeutic interventions like polypectomy, it is technically demanding in the pediatric population and often inaccessible in public health settings [9].

Consequently, the decision to proceed with diagnostic videolaparoscopy was not merely a result of resource limitations but a definitive clinical strategy to ensure both diagnosis and treatment in a single setting. This case underscores a critical clinical lesson: the possibility of small bowel polyps must be actively considered in pediatric patients presenting with unexplained severe malnutrition and cyclical vomiting, even when standard imaging is unrevealing. It reaffirms the essential role of surgical exploration as a diagnostic and therapeutic cornerstone when non-invasive modalities fail to elucidate the cause of significant gastrointestinal decline.

## Conclusions

The diagnosis of a juvenile hamartomatous polyp in the jejunum represents a significant clinical challenge due to its rarity and the nonspecific nature of the resulting symptoms. In this case, the polyp acted as a lead point for intermittent intussusception, which explained the patient’s cyclical vomiting and severe malnutrition. The successful surgical intervention and subsequent weight gain confirm that diagnostic surgical exploration serves as a critical escalation step when standard modalities - specifically upper endoscopy and colonoscopy - fail to identify a cause for persistent gastrointestinal distress.

This rare clinical scenario underscores the need for pediatric specialists to maintain a high index of suspicion for small bowel polyps even when colonoscopies and endoscopies are normal. Furthermore, the possibility of non-syndromic intestinal polyposis in such patients mandates long-term monitoring to promptly identify the emergence of potential new lesions.
